# Turtle Functions Downstream of Cut in Differentially Regulating Class Specific Dendrite Morphogenesis in *Drosophila*

**DOI:** 10.1371/journal.pone.0022611

**Published:** 2011-07-21

**Authors:** Mikolaj J. Sulkowski, Srividya Chandramouli Iyer, Mathieu S. Kurosawa, Eswar Prasad R. Iyer, Daniel N. Cox

**Affiliations:** 1 School of Systems Biology, George Mason University, Manassas, Virginia, United States of America; 2 Krasnow Institute for Advanced Study, George Mason University, Fairfax, Virginia, United States of America; Columbia University, United States of America

## Abstract

**Background:**

Dendritic morphology largely determines patterns of synaptic connectivity and electrochemical properties of a neuron. Neurons display a myriad diversity of dendritic geometries which serve as a basis for functional classification. Several types of molecules have recently been identified which regulate dendrite morphology by acting at the levels of transcriptional regulation, direct interactions with the cytoskeleton and organelles, and cell surface interactions. Although there has been substantial progress in understanding the molecular mechanisms of dendrite morphogenesis, the specification of class-specific dendritic arbors remains largely unexplained. Furthermore, the presence of numerous regulators suggests that they must work in concert. However, presently, few genetic pathways regulating dendrite development have been defined.

**Methodology/Principal Findings:**

The *Drosophila* gene *turtle* belongs to an evolutionarily conserved class of immunoglobulin superfamily members found in the nervous systems of diverse organisms. We demonstrate that Turtle is differentially expressed in *Drosophila* da neurons. Moreover, MARCM analyses reveal Turtle acts cell autonomously to exert class specific effects on dendritic growth and/or branching in da neuron subclasses. Using transgenic overexpression of different Turtle isoforms, we find context-dependent, isoform-specific effects on mediating dendritic branching in class II, III and IV da neurons. Finally, we demonstrate via chromatin immunoprecipitation, qPCR, and immunohistochemistry analyses that Turtle expression is positively regulated by the Cut homeodomain transcription factor and via genetic interaction studies that Turtle is downstream effector of Cut-mediated regulation of da neuron dendrite morphology.

**Conclusions/Significance:**

Our findings reveal that Turtle proteins differentially regulate the acquisition of class-specific dendrite morphologies. In addition, we have established a transcriptional regulatory interaction between Cut and Turtle, representing a novel pathway for mediating class specific dendrite development.

## Introduction

Neuronal dendrites occur in a staggering array of morphological conformations ranging from short, singular processes to large, highly complex structures. As dendrites form the vast majority of the post-synaptic structure, the architecture of dendritic arbors largely determines the synaptic connectivity of neuronal networks [1]. In fact, dendritic arbors have been shown to undergo dynamic remodeling in response to electrochemical signaling, which could represent a morphological correlate of cognitive processes [2]–[4]. Furthermore, the shape of dendrites alters the cable properties of the neuron, providing a mechanism for further modulation of electrochemical signaling [5], [6]. Although it is known that the spatial distribution of dendritic geometries follows certain well-described principles [7], the molecular interactions governing dendrite development remain largely unknown.

*Drosophila* dendritic arborization (da) neurons provide an exceptional model to study dendrite morphogenesis as they grow elaborate dendritic arbors that occupy a nearly two-dimensional space directly beneath the larval cuticle [8]. Investigations using da neurons as a model system have revealed a vast array of molecular mechanisms governing class specific dendrite development and dendritic field specification [9], [10]. Despite having a similar profile of cell-fate selector genes [11], [12] these da neurons can be subdivided into four unique morphological classes based on distinct patterns of dendritic arborization [8]. The diversity of da neuron dendritic arbors suggests that each class may have a unique profile of molecules and signaling pathways at work producing the characteristic morphologies. For example, the class specific distribution of the transcription factors Cut and Knot partially explains the morphological differences observed between class III and class IV da neurons by differentially regulating the actin- and tubulin-based cytoskeleton [13]–[15].

Immunoglobulin superfamily (IgSF) genes encode a large family of evolutionarily conserved proteins that function as cell-adhesion molecules, ligands, and receptors [16], [17]. IgSF molecules have been directly implicated in regulating both axonal guidance and dendritic arborization. For example, the receptor Roundabout (Robo) prevents axons from crossing the CNS midline by detecting the soluble ligand Slit, which is secreted by midline cells [18]. Moreover, a number of studies have demonstrated roles for the IgSF receptors Robo and Frazzled/Deleted in Colorectal Cancer (DCC) in mediating the development of dendrites in both PNS and CNS neurons in *Drosophila* [19]–[22]. In addition, several recent studies have demonstrated a requirement of the IgSF member Dscam in mediating dendritic self-avoidance, a form of dendritic tiling, in both *Drosophila* [23]–[25] and mouse [26].

The *Drosophila* gene *turtle* (*tutl*) encodes an evolutionarily conserved member of the Tutl/Dasm1/IgSF9 subfamily of IgSF proteins. Previous studies found that *tutl* is required for bilateral coordinated movement, however no evident defects were observed with respect to CNS morphologies [27]. Recent work has identified additional roles for *tutl* in the specification of axon and dendrite morphology. Specifically, *tutl* was reported to function in dendritic and axonal self-avoidance [28], [29] and also in proper targeting of axon projections in the CNS [30]. Analyses of the murine Tutl homolog, Dasm1, have revealed specific expression in the developing hippocampus, however loss of function studies have generated conflicting results. RNAi-based studies implicate Dasm1 in mediating dendritic arborization and synapse maturation [31], [32], whereas in Dasm1 knockout mice no evident defects in dendrite development or synaptogenesis were observed potentially due to functional redundancy of Dasm1 with the highly related *IgSF9b* gene [33].

Here, we describe novel functional roles for *tutl* in the development of class specific da neuron dendritic morphologies. Consistent with previous studies [28], our analyses revealed expression of Tutl in all da neuron subclasses with localization to cell bodies, dendrites, and axons, however, we further demonstrate differential expression levels of Tutl among da neuron subclasses. Loss of function analyses revealed differential and cell autonomous requirements for *tutl* in mediating aspects of class specific da neuron dendrite morphogenesis. In class III and IV da neurons which exhibit more complex dendritic arbors, loss of *tutl* primarily resulted in a significant decrease in total dendritic length and dendritic field coverage, whereas in class II da neurons, which exhibit simpler dendritic arbors, loss of *tutl* primarily resulted in a significant decrease in branch number. A previous study [28] reported defects in class I da neuron branching and in dendritic self-avoidance in class IV da neurons. In sharp contrast to these findings, our analyses reveal that *tutl* is largely dispensable for class I da neuron dendrite morphogenesis via MARCM and trans-allelic studies, and moreover, we found no significant defects in dendritic self-avoidance among class IV da neurons. Interestingly, gain of function studies revealed different Tutl isoforms exert isoform-specific effects on terminal dendritic branching of class II, III and IV da neurons. As the Tutl expression pattern and loss of function phenotypes resemble that previously reported for the Cut homeodomain transcription factor [34], we examined the potential regulatory relationship between Tutl and Cut and found that Cut functions as a transcriptional activator of Tutl in da neurons. Finally, we demonstrate that Tutl function is required as a downstream effector of Cut-mediated regulation of da neuron dendrite development.

## Results

### Tutl is differentially expressed in da neuron subclasses

**Figure 1:**
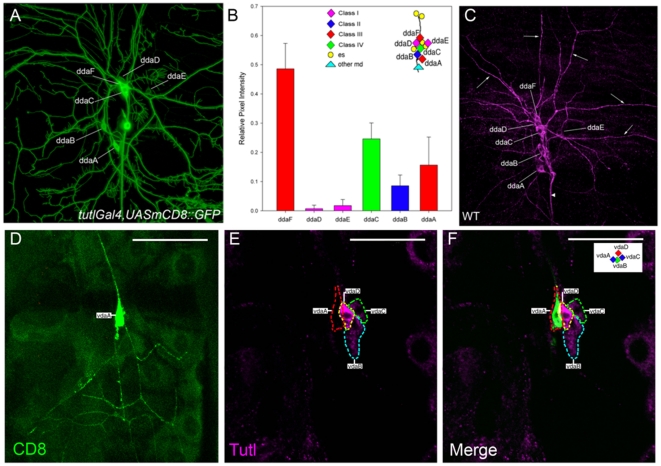
Tutl is differentially expressed in da neuron subclasses. (**A**) Neurons labeled with *tutl-GAL4,UAS-mCD8::GFP*. (**B**) Quantitative analyses of relative pixel intensity in *tutl-GAL4* transgene driver expression reveal highest levels of expression in class III and IV da neurons, respectively, moderate levels in class II da neurons, and weak levels in class I da neurons. A total of 10 dorsal cluster da neurons images labeled with *tutl-GAL4,UASmCD8::GFP* were used to performed quantitative pixel intensity analyses (n = 10). (**C**) Confocal image of wild-type third instar larvae. Third instar larval filets were dissected, fixed and labeled with rabbit anti-Tutl antibodies to visualize endogenous Tutl protein expression. The image reveals differential expression levels of Tutl protein in da neuron cell bodies with highest expression in class III (ddaF/A) and class IV (ddaC) neurons, intermediate expression in class II (ddaB) and weaker expression in class I (ddaD/E) neurons consistent with the pattern observed with the *tutl-GAL4* enhancer trap. Tutl protein expression is also observed on da neuron dendrites (arrows) and axons (arrowhead). (**D–F**) Third instar *tutl^c00018^* class II vdaA MARCM clone double labeled with rat anti-CD8 antibody (**D**) and rabbit anti-Tutl antibody (**E**). (**E,F**) Tutl staining is specifically absent from the vdaA MARCM clone, however is observed and differentially expressed in the adjacent ventral cluster da neuron cell bodies. Tutl IHC signal intensity reveals differential expression levels in da neuron subclass cell bodies with highest expression observed in the class III vdaD neuron followed by the class IV vdaB neuron and class II vdaC neuron. Tutl and CD8 signals optimized for cell body staining intensities in (E,F). Individual da neuron cell bodies are outlined by dashed lines. Size bars represent 50 µm and in all images anterior is left and dorsal up.

Previous studies first identified *tutl* as a novel IgSF member required for flight and coordinated body movements [27]. This study focused on the analysis of *tutl* expression and function in the CNS, however no detectable role was discovered for *tutl* in mediating axon pathfinding or nervous system morphogenesis [27] and thus the functional role of *tutl* in mediating these behavioral processes remains unclear. We initiated our analyses of *tutl* based on the identification of a P{GawB} enhancer trap insertion at the upstream limit of the *tutl* genomic locus (henceforth referred to a *tutl-GAL4*) which revealed a differential expression pattern in PNS da neurons. In order to quantify the *tutl* differential expression, confocal image stacks of *tutl-GAL4,UAS-mCD8::GFP* expressing da neurons were collected from the dorsal cluster of third instar larvae (Fig. 1A). We focused our analysis of Tutl expression on the dorsal cluster of da neurons owing to the fact that representative neurons from each of the four da neuron subclasses are present in this cluster. Projections of these images were then subjected to quantitative analyses of GFP expression levels via pixel densitometry. These analyses revealed the highest level of *tutl-GAL4* driven GFP expression in the dorsal-most class III da neuron (ddaF) followed by the class IV da neuron (ddaC) and the ventral-most class III da neuron (ddaA) (Fig. 1B). Intermediate levels of expression were observed in the class II da neuron (ddaB) and very low levels were observed in the two class I da neurons (ddaD and ddaE) (Fig. 1B). Our analyses further revealed *tutl-GAL4* driven GFP expression in other sensory neurons of the dorsal cluster.

To investigate the Tutl protein expression within the da neurons, immuno-histochemistry (IHC) was performed on whole-mount third instar larval filets using two distinct polyclonal anti-Tutl antibodies both of which produced nearly identical expression patterns. Consistent with the *tutl-GAL4,UASmCD8::GFP* expression pattern, immunofluorescence analyses revealed differential levels of Tutl protein expression in da neuron cell bodies with highest levels in class III and class IV da neurons, moderate levels in class II and low levels in class I da neurons (Fig. 1C,E). In addition, we observed Tutl localization to both da neuron dendrites and axons (Fig. 1C). Tutl antibody specificity was validated by the absence of Tutl protein expression in homozygous mutant *tutl^c00018^* MARCM clones (see below) (Fig. 1E,F). Moreover, these analyses indicate the *tutl^c00018^* mutation produces a protein null allele. In summary, both the *tutl-GAL4* and Tutl protein expression data consistently reveal a class specific distribution of *tutl* gene product in da neurons.

The differential expression levels of Tutl protein in da neuron subclasses is similar to that previously reported for the Cut transcription factor which has been shown to exert differential effects on class specific da neuron dendrite morphogenesis [34]. As such, we hypothesized that *tutl* may also play a functional role in mediating class specific da neuron dendrite morphogenesis. The *tutl* gene occupies a large genomic locus in which a number of available P-element insertions have been identified ranging from weak to strong hypomorphic alleles. For our study we selected a very strong, novel hypomorphic allele, *tutl^c00018^*, produced by a P{PB} insertion in the *tutl* gene. To validate the specificity of the *tutl^c00018^* allele to disruptions in the *tutl* locus, we conducted complementation analyses with the previously characterized *tutl^01085^* hypomorphic allele and with a chromosomal deficiency (*Df(2L)ed-dp*) which completely removes the *tutl* locus. These complementation analyses revealed *tutl^c00018^* specifically disrupts the *tutl* locus (Table S1). To further validate these findings, we performed rescue analyses of *tutl^c00018^* by expressing the *UAS-tutl^AT02763^* transgene under control of the pan-neuronal *elavGAL4,UAS-mCD8::GFP* driver. Consistent with previous studies in which this transgene rescued another strong *tutl* allele, *tutl^ex383^* [30], we found that this transgene completely rescued adult viability providing further evidence that the lethality observed in *tutl^c00018^* homozygous mutants is due to specific disruptions in the *tutl* locus (Table S2). The *tutl^c00018^* allele is homozygous lethal during the first larval instar, making studies using class specific *GAL4* reporters in the *tutl* mutant background difficult as da neuron dendrites have not fully elaborated until late in the third larval instar [35]. Therefore, to study the putative cell autonomous effects of disrupting *tutl* gene function, we generated homozygous mutant da neurons via the MARCM technique [36].

### Tutl functions to promote dendritic growth and branching in class III da neurons

**Figure 2:**
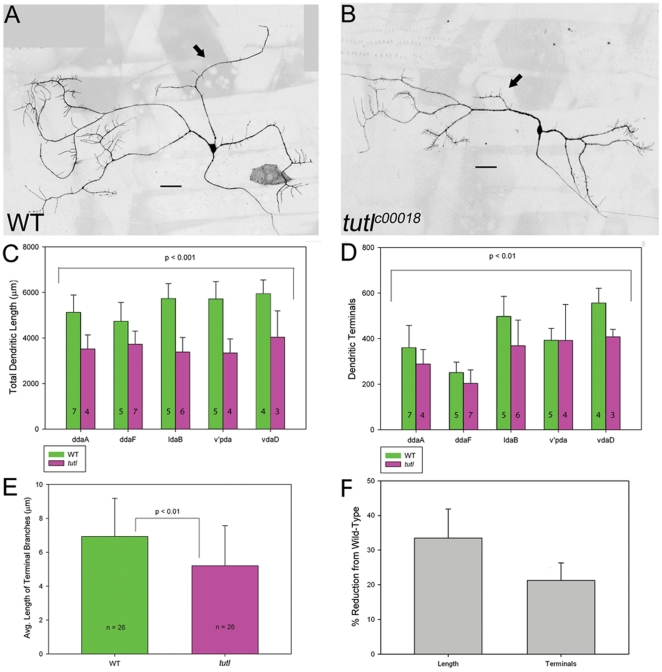
*tutl* functions cell autonomously to promote class III da neuron dendritic branching and complexity. (**A,B**) Live confocal images of representative class III da neuron MARCM clones at third larval instar. (**A**) Representative wild-type ddaA neuron. (**B**) Representative *tutl* mutant class III ddaA neuron. As compared to wild-type, *tutl* mutant class III da neurons display an overall reduction in dendritic branching, length, and field coverage. Arrows in denote correlate dendritic branches in wild-type (**A**) and *tutl* mutant (**B**) MARCM clones. Images were collected at 40X magnification in both panels and size bar represents 50 µm. (**C**) Quantitative analyses of overall dendritic length revealed a significant reduction in *tutl* mutant class III da neurons relative to wild-type controls (*p*<0.001, two way ANOVA). (**D**) Quantitative analyses of the total number of dendritic terminals revealed a significant reduction in *tutl* mutant class III da neurons relative to wild-type controls (*p*<0.01, two way ANOVA). The n value for each class III da neuron subtype for both wild-type and *tutl* mutants is indicated on the bar graph. (**E**) *tutl* is required for fully elaborating class III spine-like terminal branch protrusions. Quantitative analyses of the average length (in microns) of spine-like terminal branches reveal a significant reduction in *tutl* mutant (n = 24) class III da neurons relative to wild-type controls (n = 26) (*p*<0.01 Student's *t*-test). (**F**) Quantitative analyses revealed that in *tutl* mutants dendritic length is reduced by an average of 34% whereas terminals are reduced by 22%. For these analyses each of the five class III da neuron subtypes (ddaF, ddaA, ldaB, v'pda, vdaB) in both wild-type and *tutl* mutants were compared. The average percent reduction from wild-type in *tutl* mutants for each of the five subtypes is reported.

As we observed the highest levels of Tutl expression in class III da neurons, we initiated loss of function (LOF) phenotypic analyses focusing on this class of da neurons. Class III neurons are highly complex in terms of length and branching and are characterized by the presence of short “spine-like” terminal processes [8]. Overall, we generated a total of 24 *tutl* mutant class III da neuron clones, providing representation of each of the five characterized class III da neurons. As compared to wild-type, *tutl* mutant class III da neurons displayed a reduction in both dendritic branching complexity and field coverage (Fig. 2A,B). Overall, *tutl* mutant class III da neurons showed a significant reduction in both total dendritic length (Fig. 2C) and total number of dendritic terminals, which is a measure of dendritic branching complexity (Fig. 2D) as compared with wild-type controls. The reduction in overall dendritic length relative to wild-type averaged approximately 34% (Fig. 2F) and was characterized by a “compacted” appearance in which primary dendrites do not extend the same distance as wild-type control neurons (Fig. 2A,B). Cell autonomous *tutl* mutation also reduced the number of dendritic branches in class III neurons, as indicated by a reduction in the number of dendritic terminals. The reduction in total number of dendritic terminals relative to wild-type averaged approximately 22% in *tutl* mutant neurons and was characterized by a lower incidence of terminal branching as compared with wild-type controls (Fig. 2F). As numerous short, “spine-like” processes characterize class III neurons, we sought to determine whether the *tutl* mutation affects these fine terminal processes. To this end, we exclusively quantified the length of terminal “spine-like” branches of *tutl* mutant class III neurons and compared the results to wild-type. These analyses revealed a modest, but significant reduction in the mean length of terminal branches in *tutl* mutants (∼5 µm) as compared to wild-type controls (∼7 µm) (Fig. 2E). These results indicate that *tutl* is required for development and extension of these terminal “spine-like” protrusions.

### Tutl is required to promote dendritic growth and field coverage in class IV da neurons

**Figure 3:**
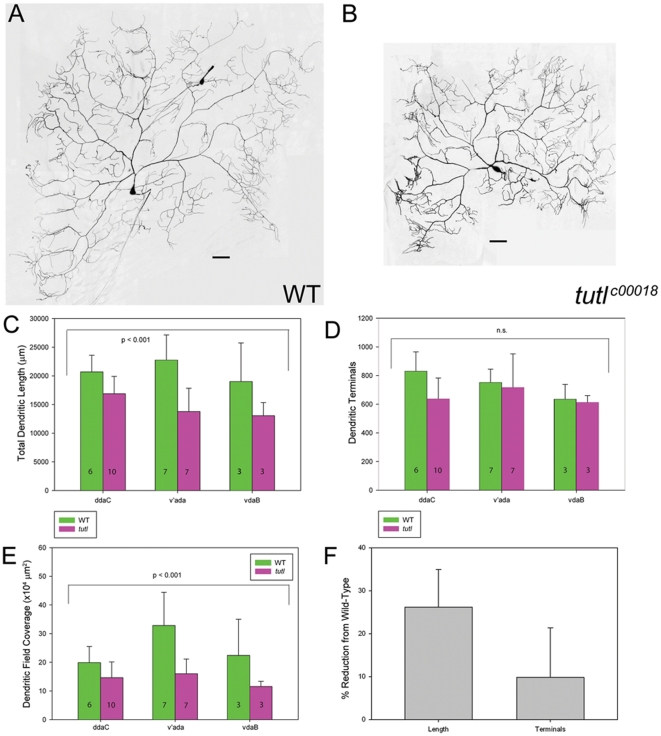
*tutl* is required for class IV da neuron dendritic growth and dendritic field specification. (**A**) Wild-type class IV v'ada neuron. (**B**) *tutl* mutant class IV v'ada neuron. As compared to wild-type, *tutl* mutant class IV da neurons display an overall reduction in dendritic length and dendritic field coverage. Images taken at 40X magnification and size bar represents 50 microns. (**C**) Quantitative analyses of overall dendritic length revealed a significant reduction in *tutl* mutant class IV da neurons relative to wild-type controls (*p*<0.001, two way ANOVA). The n value for each class IV da neuron subtype for both wild-type and *tutl* mutants is indicated on the bar graph. (**D**) Quantitative analyses of the total number of dendritic terminals revealed no significant (n.s.) reduction in *tutl* mutant class IV da neurons relative to wild-type controls (*p*>0.05, two way ANOVA). The n value for each class IV da neuron subtype for both wild-type and *tutl* mutants is indicated on the bar graph. (**E**) Quantitative analyses of total dendritic arbor demonstrate area is significantly reduced in *tutl* mutant class IV da neurons relative to wild-type controls (two way ANOVA, *p*<0.001). The n value for each class IV da neuron subtype is indicated on the bar graph. (**F**) Quantitative analyses revealed that in *tutl* mutants dendritic length is reduced by an average of 26% whereas terminals are reduced by approximately 10%. For these analyses each of the three class IV da neuron subtypes (ddaC, v'ada, and vdaB) in both wild-type and *tutl* mutants were compared. The average percent reduction from wild-type in *tutl* mutants of each of the three subtypes is reported.

Based upon the high levels of Tutl expression in ddaC and other class IV da neurons, MARCM analyses were conducted to investigate the potential function of *tutl* in mediating class IV dendrite morphogenesis. Class IV da neurons are the largest and most complex of the da neurons in terms of the number of dendritic branches, field coverage and total dendritic length [8]. A total of 20 *tutl* mutant class IV da neuron MARCM clones were generated, providing representation of each of the three characterized class IV da neurons. As compared to wild-type, *tutl* mutant class IV da neurons display a reduction in dendritic length and dendritic field coverage. Qualitatively, *tutl* mutant class IV neurons appeared smaller than wild-type, with shorter dendritic branches (Fig. 3A,B). Overall, *tutl* mutant class IV da neuron clones showed a significant reduction in total dendritic length (Fig. 3C), however the number of dendritic terminals was not significantly different between *tutl* mutant and wild-type MARCM clones (Fig. 3D). The average reduction in overall dendritic length among all subtypes of *tutl* mutant class IV neurons was approximately 26% and was characterized by reduced dendritic field coverage relative to wild-type controls (Fig. 3F). To further quantify the nature of the reduction in overall dendritic length in *tutl* MARCM clones, dendritic field area and branch order analyses were conducted. The total area covered by the dendritic arbor, as measured by the polygon method [8], was significantly decreased in *tutl* mutant neurons compared with wild-type neurons (Fig. 3E). Branch order analysis revealed some additional aspects of dendritic arbor change. *tutl* mutant class IV da neurons showed a proximally shifted distribution of branch order relative to the cell body characterized by an increased percentage of lower-order branches as compared with wild-type (Fig. S1). This indicates that *tutl* is required for the formation of higher-order branches. Therefore, *tutl* appears to be required for the establishing full dendritic field coverage in class IV da neurons.

Previous analyses have reported defects in class IV da neuron dendritic self-avoidance among *tutl* mutants [28]. To assess whether defects in iso-neuronal avoidance among class IV neurons were also observed in our studies, we quantified the number of dendritic crossovers in *tutl^c00018^* MARCM clones relative to wild-type controls (Fig. S2). These analyses failed to reveal any statistically significant difference between *tutl* mutant and wild-type class IV da neurons with respect to the average number of crossovers between iso-neuronal dendrites (Fig. S2A). As *tutl^c00018^* mutant class IV da neurons were shown to produce a significant reduction in overall dendritic length and field coverage, we normalized the number of iso-neuronal dendrite crossovers to total dendritic length for both *tutl^c00018^* mutant and wild-type class IV da neuron MARCM clones. Despite this normalization, our analyses revealed no statistically significant difference between *tutl* mutants and wild-type controls with respect to the average number of crossovers/µm for iso-neuronal dendrites (Fig. S2B).

### Tutl is required to promote dendritic branching and extension in class II da neurons

**Figure 4:**
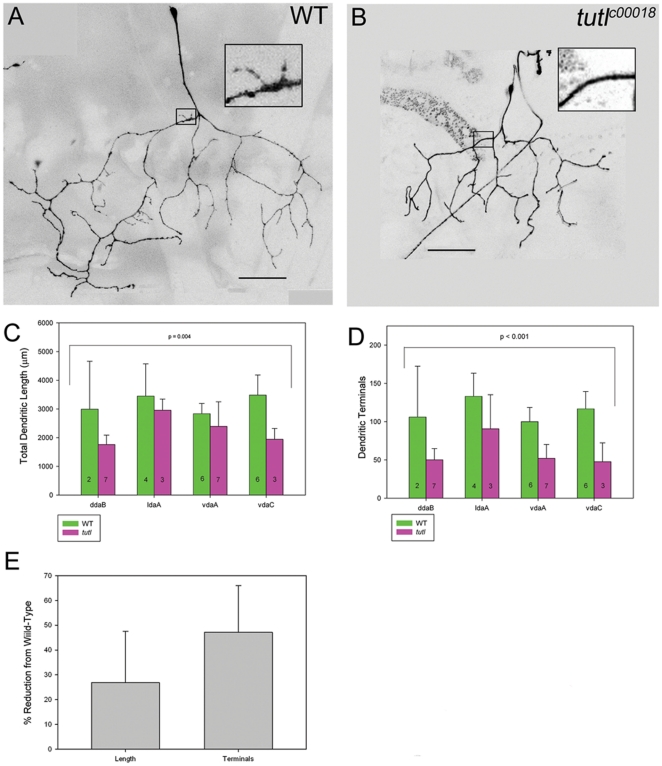
*tutl* is required to promote both dendritic branching and extension in class II da neurons. (**A**) Wild-type class II vdaA neuron. Note terminal branching complexity in inset. (**B**) *tutl* mutant class II vdaA neuron. As compared to wild-type, *tutl* mutant class II da neurons display an overall reduction in dendritic branching particularly with respect to terminal branching and to a lesser extent overall dendritic length. Note loss of terminal dendritic branches in inset. Images taken at 40X magnification and size bar represents 50 microns. (**C**) Quantitative analyses of overall dendritic length revealed a significant reduction in *tutl* mutant class II da neurons relative to wild-type controls (two way ANOVA, *p*<0.001). The n value for each class II da neuron subtype for both wild-type and *tutl* mutants is indicated on the bar graph. (**D**) Quantitative analyses of the total number of dendritic terminals revealed a significant reduction in *tutl* mutant class II da neurons relative to wild-type controls (two way ANOVA, *p*<0.001). The n value for each class II da neuron subtype for both wild-type and *tutl* mutants is indicated on the bar graph. (**E**) Quantitative analyses revealed an average 27% reduction in overall dendritic length relative to an average 46% reduction in the number of dendritic terminals in *tutl* mutant class II da neurons relative to wild-type controls. For these analyses each of the four class II da neuron subtypes (ddaB, ldaA, vdaA, vdaC) in both wild-type and *tutl* mutants was compared. The average percent reduction from wild-type in *tutl* mutants of each of the four subtypes is reported.

Based upon the intermediate levels of *tutl* expression in class II da neurons, MARCM analyses were conducted to investigate the potential function of *tutl* in mediating class II da neuron dendrite morphogenesis. To this end, a total of 20 *tutl* mutant class II da neuron MARCM clones were generated, providing representation of all four class II da neuron subtypes. Class II da neurons have some morphological features in common with both class I and class III da neurons. Similar to class I neurons, they have few primary branches, whereas like class III neurons, they have short “spine-like” processes, although fewer in number. As compared to wild-type, *tutl* mutant class II da neurons display an overall reduction in dendritic branching complexity and dendritic length (Fig. 4). In *tutl* mutants, class II da neurons became smaller, and displayed a reduced number of short terminal branches (Fig. 4A,B).

Overall, *tutl* MARCM class II da neuron clones showed a significant reduction in both total dendritic length (Fig. 4C) and total number of dendritic terminals (Fig. 4D) as compared to wild-type MARCM clones. Although *tutl* mutant class II da neurons displayed a reduction in both length and branching, the reduction in dendritic terminals was objectively greater than the reduction in dendritic length. The reduction in overall dendritic length relative to wild-type averaged approximately 27% in *tutl* mutant neurons whereas the reduction in the total number of dendritic terminals relative to wild-type averaged nearly 46% (Fig. 4E).

Collectively, analyses of class II-IV da neurons revealed a phenotypic pattern in terms of *tutl* function in mediating da neuron dendrite morphogenesis. In morphologically simple classes of da neurons, such as class II, mutations in *tutl* caused more of a decrease in dendritic branching, whereas in more complex classes of da neurons, such as class III and IV, mutations in *tutl* caused more of a decrease in dendritic length. These results support class-specific functions for *tutl*: promoting dendritic branching in simpler, less-branched neurons and promoting dendritic extension in more complex highly branched neurons.

### *tutl* is largely dispensable for class I da neuron dendrite morphogenesis

**Figure 5:**
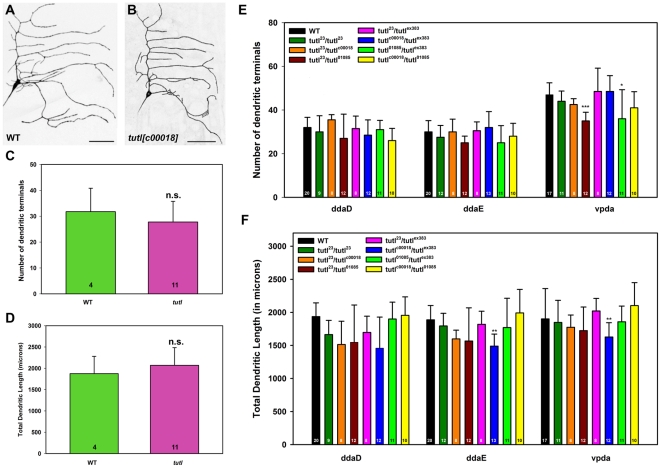
*tutl* is largely dispensable for class I da neuron dendrite morphogenesis. (**A,B**) Live confocal images of representative class I ddaE neuron MARCM clones at the third larval instar. (**A**) Representative wild-type (WT) ddaE neuron. (**B**) Representative *tutl^c00018^* mutant class I ddaE neuron. As compared to wild-type, *tutl^c00018^* mutant class I da neurons display no qualitative defects in overall dendritic branching or extension. Images were collected at 40X magnification in both panels and size bar represents 50 microns. (**C**) Quantitative analyses of overall number of dendritic terminals revealed no significant (n.s.) difference between wild-type and *tutl* mutants (Mann-Whitney rank-sum test, *p*>0.05). (**D**) Quantitative analyses of overall dendritic length revealed no significant difference between wild-type and *tutl* mutants (Mann-Whitney rank-sum test, *p*>0.05). The *n* value for both wild-type and *tutl* mutants is indicated on the bar graph. (**E**) Quantitative pairwise analyses of total number of dendritic terminals revealed no significant statistical difference between wild-type and trans-allelic *tutl* mutant class I ddaD and ddaE neurons (*p*>0.05, Student's *t* test) labeled by *GAL4^221^,UAS-mCD8::GFP*. In vpda neurons, a significant reduction in the number of dendritic terminals was observed in only two trans-allelic combinations. (**F**) Quantitative pairwise analyses of total dendritic length generally revealed no significant statistical difference (*p*>0.05, Student's *t* test) between wild-type and trans-allelic *tutl* mutant class I da neurons with the exception of *tutl^c00018^/tutl^ex383^* where there was a significant reduction in length in ddaE and vpda neurons. The total *n* value for each genotype quantified is reported on the bar graphs. Only statistically significant *p* values are reported on the graph as follows (* = *p*<0.05; ** = *p*<0.01; *** = *p*<0.001) and all other pairwise analyses were not significant.

Previous studies suggested that *tutl* is required to restrain dendrite branching in class I da neurons [28]. To determine if this phenotype was observed in *tutl^c00018^* mutants, we conducted MARCM LOF analyses in class I neurons. In contrast to previous findings, our analyses revealed no qualitative or statistically significant quantitative defects in class I da neuron dendrite morphologies in *tutl^c00018^* mutant clones relative to wild-type controls (Fig. 5A–D). This phenotypic difference, however, does not appear to result from any residual Tutl protein from precursor cells persisting in the MARCM clones as we have shown *tutl^c00018^* MARCM clones have no detectable Tutl protein expression (Fig. 1E). As these findings stand in sharp contrast to previous reports [28], we conducted additional trans-allelic studies between *tutl^c00018^* and three other previously characterized *tutl* alleles [27], [28], [30] to determine if the phenotypes observed were due to allelic variation. In total, we examined seven allelic combinations, including analyses of the previously reported *tutl^23^* mutant allele which had been implicated in disrupting class I dendrite morphology. Moreover, we extended previous analyses [28] by performing systematic studies of all three class I da neurons (ddaD, ddaE, and vpda). In general these trans-allelic studies failed to reveal any significant defects in either the number of dendritic terminals (Fig. 5E) or total dendritic length (Fig. 5F) among individual class I da neuron subtypes. In those isolated allelic combinations where we did observe an effect on either terminals or length, the effects were a reduction relative to controls which is again in opposition to previously reported results [28] (Fig. 5E,F). Importantly, we were unable to reproduce the results reported by Long *et al.* (2009) despite using the same alleles and allelic combinations in any of the class I da neurons. Collectively, these analyses strongly indicate that *tutl* is largely dispensable for class I da neuron dendrite morphogenesis, and in the limited trans-allelic combinations where an effects was observed, the data suggest, if any requirement, that *tutl* promotes, rather than restrains, class I morphogenesis.

### Isoform-specific Tutl overexpression differentially regulates dendrite branching

The *tutl* gene encodes multiple protein isoforms including both membrane-bound and soluble, potentially secreted isoforms [28], [30]. In a previous study [28] overexpression of the longest known membrane bound Tutl isoform, RE40452, resulted in an inhibition of dendrite branching in class IV da neurons, however no effect was reported following overexpression of this single Tutl isoform in class I-III da neurons. To assess the potential of distinct, isoform-specific Tutl function in mediating dendritic branching, we examined overexpression of two potentially secreted isoforms, AT02763 and GH15753, as well as two membrane bound isoforms, HL01565 and LD28224, in class I-IV da neurons. Each of these Tutl isoforms, including the previously described RE40452 [28], has been demonstrated to encode functional Tutl proteins capable of rescuing reported null *tutl* alleles [28], [30].

**Figure 6:**
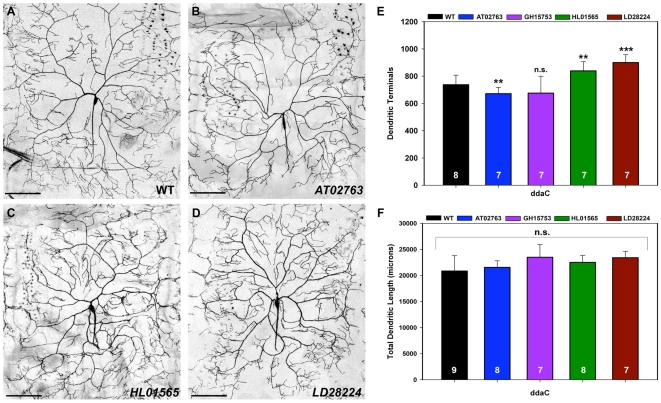
Isoform-specific Tutl overexpression differentially effects dendrite branching in class IV da neurons. (**A–D**) Representative live confocal images of wild-type and Tutl isoform overexpressing class IV ddaC neurons collected at 20X magnification. Anterior is left and dorsal up and size bar represents 100 microns. (**A**) Wild-type (WT) class IV ddaC neuron. (**B**) Overexpression of the non-membrane bound Tutl AT02763 isoform reduces dendritic branching in ddaC neurons particularly at dendritic terminals. (**C,D**) Overexpression of the membrane bound Tutl HL01565 (C) and Tutl LD28224 (D) isoforms increases dendritic branching in ddaC neurons most evident at dendritic terminals. (**E**) Quantification of ddaC dendritic branch termini revealed a statistically significant decrease with AT02763 overexpression and increase with both HL01565 and LD28224 overexpression relative to wild-type controls. (**F**) Quantification of ddaC total dendritic length revealed no statistically significant (n.s, *p*>0.05) change with Tutl isoform overexpression relative to wild-type controls. The total *n* value for each genotype quantified is reported on the bar graphs. Statistically significant *p* values are reported on the graph as follows (** = *p*<0.01; *** = *p*<0.001; n.s. = not significant). Genotypes (A–D): **WT**: *GAL4^477^,UASmCD8::GFP/+*; **AT02763**: *UAS-tutl^AT02763^/+; GAL4^477^,UASmCD8::GFP/+*; **GH15753**: *UAS-tutl^GH15753^/+; GAL4^477^,UASmCD8::GFP/+*; **HL01565**: *GAL4^477^,UASmCD8::GFP/+*; *UAS-tutl^HL01565^/+*; **LD28224**: *GAL4^477^,UASmCD8::GFP/+*; *UAS-tutl^LD28224^/+*.

**Figure 7:**
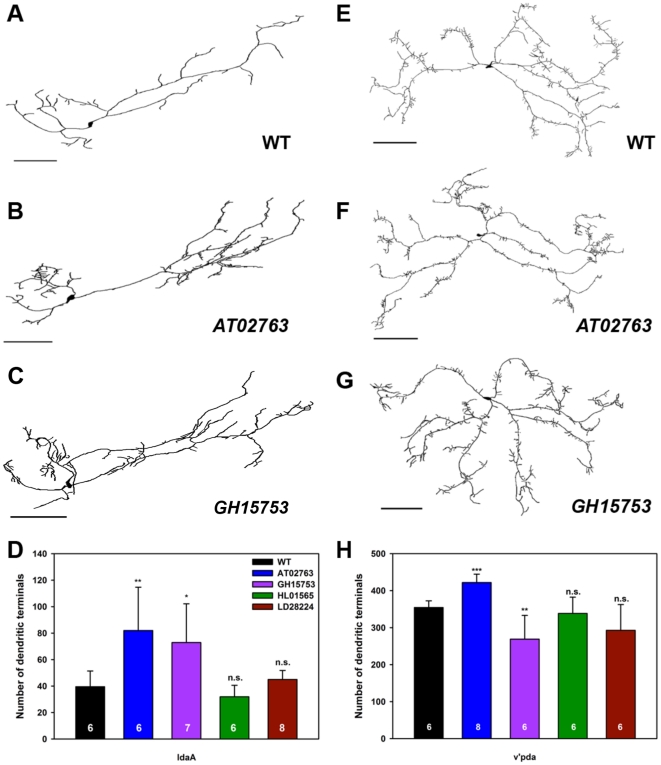
Isoform-specific Tutl overexpression differentially regulates dendrite branching in class II and III da neurons. Representative tracings of class II ldaA (**A–C**) and class III v'pda (**E–G**) neurons from third instar larvae. Anterior is left and dorsal up and size bars represents 150 microns for ldaA and 100 microns for v'pda. (**A**) Wild-type (WT) ldaA neuron. Overexpression of the non-membrane bound Tutl AT02763 (**B**) and GH15753 (**C**) isoforms increased terminal dendritic branching in ldaA neurons. (**D**) Quantitative analyses of dendritic branching revealed a statistically significant increase with AT02763 and GH15753 overexpression relative to wild-type controls. (**E**) Wild-type (WT) v'pda neurons. Overexpression of the Tutl AT02763 isoform (**F**) increased terminal dendritic branching, whereas overexpression of the Tutl GH15753 isoform (**G**) decreased terminal dendritic branching in v'pda neurons. (**H**) Quantitative analyses of dendritic branching revealed a statistically significant increase with AT02763 and decrease with GH15753 overexpression relative to controls. The total *n* value for each genotype quantified is reported on the bar graph. Statistically significant *p* values are reported on the graph as follows (* = *p*<0.05; ** = *p*<0.01; *** = *p*<0.001; n.s. = not significant). Genotypes (A–H): **WT**: *GAL4^C161^,UASmCD8::GFP/+*; **AT02763**: *UAS-tutl^AT02763^/+;*+;*GAL4^C161^,UASmCD8::GFP/+*; **GH15753**: *UAS-tutl^GH15753^/+;*+; *GAL4^C161^,UASmCD8::GFP/+*; **HL01565**: *UAS-tutl^HL01565^/GAL4^C161^,UASmCD8::GFP*; **LD28224**: *UAS-tutl^LD28224^/GAL4^C161^,UASmCD8::GFP*.

In class IV da neurons (ddaC), overexpression of non-membrane bound AT02763 isoform led to a inhibition of dendritic branching, consistent with previous observations for RE40452 [28], relative to wild-type controls (Fig. 6B,E). In contrast, overexpression of the membrane bound HL01565 (Fig. 6C) and LD28224 (Fig. 6D) isoforms promoted dendritic branching (Fig. 6E) relative to wild-type controls. Quantitative analyses of overall dendritic length however revealed no significant effect following overexpression of any of the four Tutl isoforms examined relative to control suggesting that the predominant effect on dendritic branching was at the level of the fine terminal branches of class IV da neurons (Fig. 6F). To analyze putative isoform-specific effects on class II and III neurons, we used the class I-III *GAL4^C161^,UAS-mCD8::GFP* driver. In contrast to class IV da neurons, overexpression of the soluble AT02763 and GH15753 isoforms in class II da neurons (ldaA) resulted in increased terminal dendritic branching relative to controls, whereas overexpression of the membrane-bound HL01565 and LD28224 isoforms had no significant effect on class II dendritic branching (Fig. 7A–D). Moreover, quantification of total dendritic length revealed no significant change with overexpression of any of the isoforms (data not shown) indicating the primary effect was at the level of dendritic branching. In class III da neurons (v'pda), overexpression of AT02763 led to an increase in terminal dendritic branching, whereas overexpression of GH15753 caused a decrease in terminal branching relative to controls (Fig. 7E–H). No significant effects on dendritic branching were observed with overexpression of HL01565 or LD28224 (Fig. 7H) nor did we observe any significant change in overall dendritic length of v'pda neurons with any of the isoforms (data not shown). These isoform-specific results contrast with previous analyses of the RE40452 isoform in which no dendritic defects were reported upon overexpression in class II or III da neurons [28]. In contrast to the isoform-specific overexpression effects observed in class II-IV da neurons, dendritic branching and overall dendritic length were unaffected in class I da neurons relative to controls consistent with previous findings for RE40452 [28] (Fig. S3). Collectively, these data indicate that Tutl can exert complex, context-dependent, isoform-specific effects on class-specific da neuron dendrite branching.

### Cut specifically binds to the *tutl* promoter and positively regulates Tutl expression

**Figure 8:**
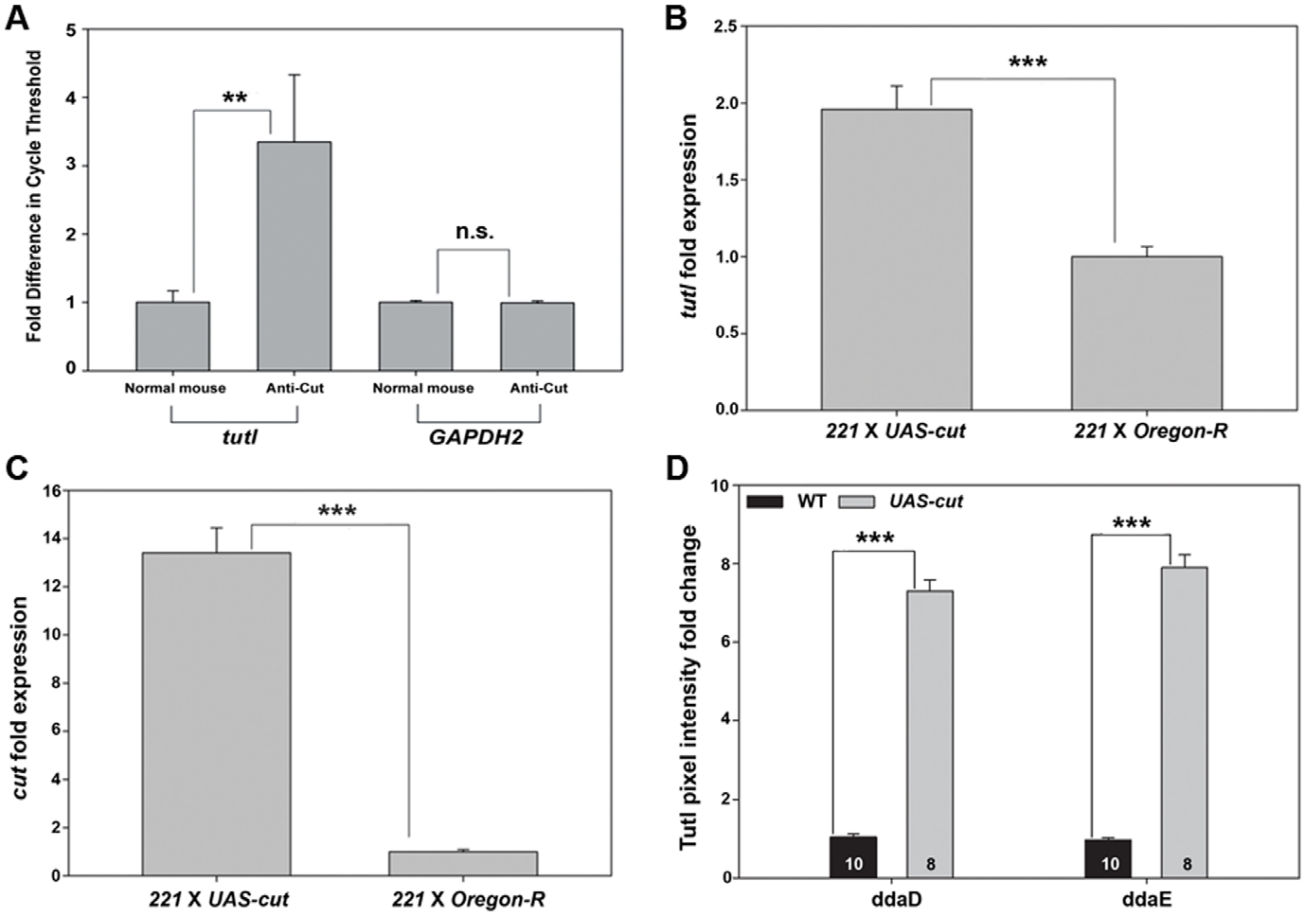
Cut specifically binds to the *tutl* promoter and positively regulates Tutl expression. (**A**) qPCR analysis reveals a significant 3.35 ± 1.26 fold increase in *tutl* genomic DNA in chromatin immunoprecipitated with a monoclonal anti-Cut antibody as compared to the normal mouse IgG control (n = 4). As a negative control, *GAPDH2* levels are unchanged between anti-Cut and mouse IgG preparations (n = 4). (**B–C**) qPCR of RNA isolated from either wild-type (*221* X *Oregon-R*) or Cut overexpressing (*221* X *UAS-cut*) class I da neurons shows that in Cut overexpressing class I da neurons *tutl* mRNA is significantly 1.96 fold overexpressed compared with wild type class I neurons (**B**) (n = 4). *cut* mRNA was present at a significant 13.39 fold increase in Cut overexpressing class I da neurons compared with wild-type (**C**) (n = 4). (**D**) Quantitative mean pixel density analyses of Tutl protein expression revealed a 7–8 fold increase in Tutl immunoreactivity levels in class I neurons overexpressing *UAS-cut* (n = 8) relative to wild-type controls (n = 10). Statistically significant *p* values are reported on the graphs as follows (** = *p*<0.01; *** = *p*<0.001; n.s. = not significant). Genotypes: (**B,C**) *221* X *Oregon-R* corresponds to *GAL4^221^,UAS-mCD8::GFP/+*; *221* X *UAS-cut* corresponds to *UAS-cut/+;GAL4^221^,UAS-mCD8::GFP/+*. (**D**) WT corresponds to *GAL4^221^,UAS-mCD8::GFP/+*; *UAS-cut* corresponds to *UAS-cut/+; GAL4^221^,UAS-mCD8::GFP/+*.

The Tutl expression pattern in da neurons is very similar to that of another gene that is a known regulator of class specific da neuron dendrite morphology, the homeodomain transcription factor *cut* [34]. We also noted that some of the phenotypes observed in *tutl* LOF class III and IV are similar to those reported for LOF *cut* [34], [15]. Furthermore, analyses of the *tutl* promoter region positively identified a precisely conserved upstream regulatory element (URE) sequence (5′-AATCAAAC-3′) beginning at nucleotide position 281 of the *tutl* genomic locus (-200 nt from the common transcriptional start site of all known *tutl* isoforms). This URE sequence has previously been found to represent a Cut transcriptional binding site [37]. These data suggest the potential of a regulatory relationship between *cut* and *tutl*. To investigate this possible transcriptional regulation, we conducted chromatin immunoprecipitation (ChIP) assays to evaluate whether the Cut protein could specifically bind to the genomic *tutl* enhancer *in vitro*. For these analyses, isolated crosslinked chromatin from *Drosophila* S2 cells was used for immunoprecipitation using a monoclonal anti-Cut antibody (2B10). qPCR was employed using primers to amplify a region of the *tutl* promoter region containing the URE site. qPCR analyses of the immunoprecipitated chromatin revealed the presence of *tutl* genomic DNA greater than three-fold higher than that observed with the normal mouse IgG antibody control (Fig. 8A). As a negative control, primers against the promoter region of *GAPDH2*, which lacks any Cut consensus transcriptional binding sites, were tested on immunoprecipitated chromatin and revealed no significant difference between anti-Cut and normal IgG preparations (Fig. 8A).

This experiment demonstrated that the Cut protein can bind specifically to the *tutl* promoter *in vitro*, however, whether Cut may act as a transcriptional activator or repressor *in vivo* remained uncertain. Consistent with previous studies [28], we found that Tutl antibody staining was present in *cut* mutant da neuron MARCM clones (data not shown). These results indicate that Tutl expression in da neurons is not solely dependent upon Cut transcriptional regulation, however this does not rule out the possibility that Cut may act to regulate Tutl expression in these neurons. To explore this question, we performed magnetic bead-based cell sorting [38] to isolate class I da neurons ectopically overexpressing Cut. The RNA obtained from these purified cells was then used to perform qPCR using primers for both *tutl* and *cut* mRNA. The qPCR results demonstrated that in Cut overexpressing class I da neurons, *tutl* mRNA is present two-fold higher than in wild-type controls (Fig. 8B). As a positive control, the level of *cut* mRNA was also evaluated following overexpression by qPCR which revealed a thirteen-fold upregulation as compared with wild-type controls (Fig. 8C). To further validate the results of the qPCR, we performed IHC analyses of Tutl expression in class I da neurons ectopically overexpressing Cut. As compared to wild-type, we observed a significant increase (7–8 fold) in Tutl immunoreactivity in class I da neurons overexpressing Cut (Fig. 8D). Collectively, these results demonstrate that Cut can bind to the *tutl* promoter region and transcriptionally upregulate *tutl* mRNA and protein expression *in vivo*.

### Tutl functions as a downstream effector of Cut-mediated regulation of da neuron dendrite morphogenesis

**Figure 9:**
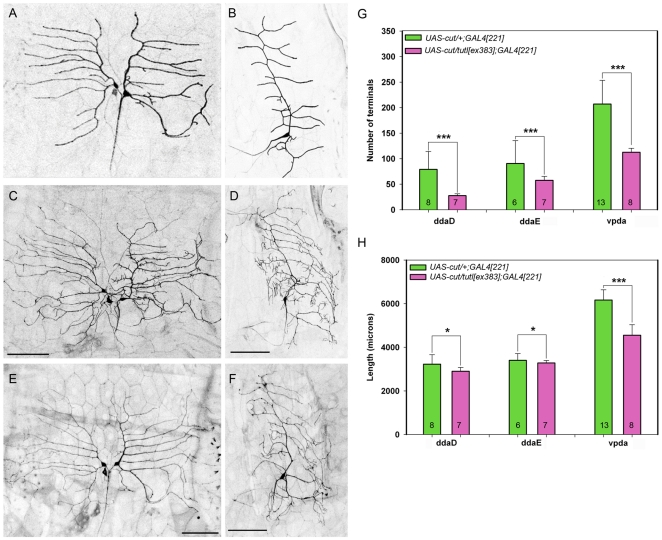
Tutl functions as a downstream effector of Cut-mediated regulation of da neuron dendrite development. (**A–F**) Live confocal images of representative class I da neurons (ddaD, ddaE, vpda) at the third larval instar. Images were collected at 20X magnification and size bars represents 100 microns. (**A,B**) Representative images of wild-type dorsal ddaD/E neurons (A) and ventral vpda neuron (B) ectopically overexpressing Cut. (**C,D**) Representative images of dorsal ddaD/E neurons (C) and ventral vpda neuron (D) ectopically overexpressing Cut. Note, ectopic overexpression of Cut results in class I neurons displaying increased dendritic branching complexity characterized by a high incidence of short dendritic protrusions emanating from the primary branches as compared to wild-type. (**E,F**) Representative images of dorsal ddaD/E neurons (E) and ventral vpda neuron (F) in which ectopic overexpression of Cut is combined with the *tutl^ex383^* allele. As compared to Cut overexpression alone, introduction of one copy of the *tutl^ex383^* mutant allele results in strong suppression of the Cut phenotype particularly with respect to the incidence of short dendritic protrusions. (**G**) Quantitative analyses reveal that the total number of dendritic terminals is significantly reduced in animals carrying one copy of the *tutl^ex383^* mutant allele relative to Cut ectopic overexpression alone. (**H**) Quantitative analyses reveal a modest, yet statistically significant, decrease in overall dendritic length in animals carrying one copy of the *tutl^ex383^* mutant allele relative to Cut ectopic overexpression alone. The total *n* value for each neuron subtype is indicated on the bar graph. Statistically significant *p* values are reported on the graphs as follows (* = *p*<0.05; *** = *p*<0.001). Genotypes: (**A,B**) *GAL4^221^,UASmCD8::GFP/+*; (**C,D**) *UAS-cut/+; GAL4^221^,UASmCD8::GFP/+*; (**E,F**), *UAS-cut/tutl^ex383^; GAL4^221^,UASmCD8::GFP/+*.

To determine the potential functional significance of Cut transcriptional regulation of Tutl expression, we examined whether Tutl was required to mediate previously described effects of Cut on da neuron dendrite morphogenesis [34]. Previous studies demonstrated that ectopic overexpression of Cut in class I da neurons resulted in a dramatic increase in dendritic branching complexity characterized by increased overall dendritic length and the development of numerous “spine-like” dendritic filopodia emanating from the primary dendritic branches highly similar to those normally observed in class III da neurons [34] (Fig. 9A–D). To assess the role of Tutl as a potential downstream effector in mediating the Cut overexpression phenotype, we phenotypically compared class I da neurons ectopically overexpressing Cut with class I neurons in which we ectopically overexpressed Cut and reduced *tutl* gene function by 50% via introduction of *tutl^ex383^* allele. The *tutl^ex383^* allele was selected for these analyses as it has previously been demonstrated to be a molecular and genetic null allele that is embryonically lethal and is characterized by a 11,430 bp genomic deletion which disrupts all known *tutl* isoforms [30]. We reasoned that if the ectopic Cut overexpression phenotype in class I neurons is dependent upon Tutl activation, then removing Tutl gene function by 50% may lead to a suppression of the Cut-induced phenotype. Indeed, these analyses revealed strong suppression of the Cut ectopic overexpression phenotype upon introduction of one copy of the *tutl^ex383^* allele in all class I da neurons (Fig. 9E,F). The Tutl-mediated phenotypic suppression was particularly pronounced with respect to the number of dendritic terminals in which there was an average reduction of 65% in ddaD, 36% in ddaE, and 46% in vpda relative to the average number of terminals observed in Cut ectopically overexpressing class I da neurons alone (Fig. 9G). Quantitative analyses of Tutl-mediated suppression was also statistically significant with respect to overall dendritic length, albeit to a lesser extent than the suppression effects on the number of dendritic terminals (Fig. 9H). These data reveal that Tutl functions as a downstream effector of Cut in directing da neuron dendrite development and suggest Tutl is particularly required to promote terminal dendritic branching.

## Discussion

The establishment of normal dendritic morphology is critical in the formation of functional neural networks. Using the *Drosophila* PNS as a model, this study demonstrates differential expression of Tutl in da neuron subclasses and in addition reveals class-specific roles for Tutl in mediating both branching and extension of da neuron dendritic arbors. Gain of function studies reveal isoform-specific functions for Tutl in mediating terminal dendritic branching in class II, III, and IV da neurons. Furthermore, we have identified a novel transcriptional regulatory interaction between Tutl and Cut which reveals Tutl functions as a downstream target of Cut in mediating class specific da neuron dendrite development.

### Tutl differentially regulates class specific dendrite morphogenesis

Analyses of *tutl-GAL4* and Tutl protein expression reveal differential expression levels in da neuron subclasses. The highest levels of *tutl* expression were observed in the more complex class III and IV da neurons, whereas moderate levels of Tutl expression were observed in class II da neurons, followed by weaker levels in class I da neurons. This differential expression pattern correlates with certain aspects of morphological complexity by which these neurons have been subdivided into classes [8]. Specifically, the highest levels of Tutl expression correlate with the appearance of numerous, short processes extending from longer primary branches characteristic of class III and IV da neurons. Interestingly, the transcription factor Cut, a known regulator of dendritic growth, shows a strikingly similar pattern of expression in da neurons [34]. In addition to differential Tutl expression in da neuron cell bodies, we observed Tutl localization to dendrites and axons.

Consistent with differential Tutl expression levels, we observed novel, cell autonomous and class specific defects in da neuron dendrite morphogenesis in *tutl* mutants. In class III da neurons, *tutl* mutation had objectively the most severe phenotype. A significant reduction in both branching and length was observed. Overall, class III da neurons mutant for *tutl* appeared smaller than their wild-type counterparts, with reduced branching and a reduction in number and length of their characteristic “spine-like” processes. In fact, by directly analyzing the length of terminal branches, we were able to show that *tutl* is required for full extension of these processes. MARCM analyses further revealed that class IV and class II da neurons each require Tutl for normal dendrite development. However, Tutl appears to regulate different aspects of morphogenesis in each class. For the complex, space-filling dendrites of class IV neurons, Tutl is required for dendrites to reach the appropriate length and establish full dendritic field coverage. In contrast, class II neurons require Tutl primarily to promote an appropriate number of branches.

Many of the findings reported herein sharply contrast with those of a previous study [28] in which a very similar set of experimental analyses were performed to investigate *tutl* function in da neurons. Among the differences in findings between studies is the question of *tutl* function in class I da neurons. A previous study implicated Tutl in restricting class I neuron dendritic branching complexity [28], however, in general, we find that Tutl is largely dispensable for class I branching. In light of this striking difference, we repeated the previous studies using the same *tutl^23^* mutant allele, and extended our analyses to incorporate additional novel allelic combinations and to include all three class I da neuron subtypes, however we were unable to reproduce the results of the previous study in any class I neuron. These results demonstrate that, contrary to previous findings, Tutl function is largely dispensable for class I dendrite development and moreover, indicate that allelic variation is not a contributing factor to the observed differences between studies at least for class I neurons.

In addition, we find that *tutl* exerts differential effects on class II and III dendritic branching and growth, whereas a previous study [28], using the *tutl^23^* mutant allele, fails to reveal any significant effects on class II and III dendrite development. The basis for the differences observed herein and in the previous study [28] are not entirely clear, however, distinctions between studies include the use of independent *tutl* alleles, suggesting the potential of allelic variation with respect to phenotypic effects, as well as the fact that the previous study [28] focused exclusively on analyses of the class II ddaB and class III ddaA neurons, whereas the present study examined effects on class II or III neurons as a whole incorporating data from all subtypes within an individual class. Finally, while previous reports, using the *tutl^23^* allele, suggested that Tutl was required to mediate dendritic self-avoidance in class IV da neurons [28], we were unable to detect any significant effect on self-avoidance in our study. Ultimately, the basis for the observed phenotypic differences with respect to self-avoidance remain unclear, however may again potentially be attributable to allelic variation as independent alleles were used in each study.

### Isoform-specific functions for Tutl in dendrite morphogenesis

Isoform-specific Tutl overexpression was found to differentially regulate dendrite branching in class II, III, and IV da neurons, however produced no significant defects in class I da neuron dendrite development. Consistent with these findings, a previous study [28] reported an inhibition of class IV dendrite branching upon overexpression of the membrane bound Tutl RE40452 protein isoform and observed no defects in class I dendrite development upon Tutl RE40452 overexpression. Interestingly, our results demonstrate that overexpression of one of the previously characterized non-membrane bound Tutl isoforms [30], AT02763, inhibited terminal dendritic branching in class IV ddaC neurons, whereas overexpression of two membrane-bound Tutl isoforms, HL01565 and LD28224, promoted terminal dendritic branching in ddaC neurons. Given that both membrane bound [28] and soluble isoform overexpression inhibit class IV dendrite branching, whereas two other membrane bound isoforms promote branching there is no simple relationship in terms of soluble vs. trans-membrane Tutl isoforms in regulating class IV dendritic branching. Moreover, analyses of isoform-specific overexpression in class II and III da neurons further illustrate the complexity of Tutl function in mediating class specific dendritic branching. We found that overexpression of the soluble AT02763 and GH15753 isoforms in class II neurons promotes dendritic branching, whereas the membrane bound isoforms had no effect. In contrast, in class III neurons, overexpression of AT02763 and GH15753 isoforms in class III neurons had opposing effects on dendritic branching and again overexpression of the membrane bound forms had no effect. Consistent with these isoform-specific dendritic effects, a recent study demonstrated Tutl is required for normal CNS axon development and found that specific Tutl protein isoforms function as attractive axon guidance cues and promote axon branching and invasiveness [30]. This study demonstrated that overexpression of the membrane bound Tutl isoforms, HL01565 and LD28224, produced increased branching of ISNd motor neurons and sprouting of extra axonal processes in retinal neurons [30] similar to the increased dendritic branching observed with overexpression of these Tutl isoforms in class IV da neurons. Moreover, this study demonstrated that soluble Tutl isoforms *e.g.* AT02763 can act as attractive axon guidance cues suggesting Tutl may also exhibit non-cell autonomous functions. However, overexpression of AT02763 in class IV da neurons led to inhibition of dendrite branching, suggesting a potential repulsive function for dendrite branching. Multiple functioning of a gene in both dendrite and axon growth is not without precedent. Another conserved IgSF member, the receptor Roundabout (Robo), first discovered for its regulation of axonal midline crossing [18] has recently been shown as necessary for proper dendrite growth in the CNS and PNS [19]–[21], [39]. In da neurons, Robo serves to restrict dendrite outgrowth, consistent with its role as a receptor of repulsive signals [20]. Collectively, these isoform-specific overexpression studies suggest that distinct Tutl isoforms may function in a complex context-dependent, cell-type specific manner to regulate dendritic branching morphology. It is possible that these various Tutl isoforms function in concert to “fine tune” class specific dendritic branching complexity.

### Regulation of Tutl-mediated dendrite morphogenesis

The Cut homeodomain transcription factor was among the first genes shown to regulate class specific da neuron dendrite morphogenesis [34]; however, to date the pathway through which it functions has been unknown. Although Cut was shown to influence the level of the Knot transcription factor expression [15], a direct transcriptional relationship was not established. Our studies reveal that Tutl expression is positively regulated by the Cut. We demonstrate that Cut specifically binds to the *tutl* promoter sequence and acts as a transcriptional activator of Tutl expression in da neurons thereby identifying a novel transcriptional regulatory mechanism by which Tutl may mediate class-specific dendrite morphogenesis. Moreover, genetic interaction studies reveal that Tutl functions as a downstream effector of Cut-mediated regulation of a da neuron dendrite morphogenesis.

Expression in class I da neurons, albeit at low levels, established that Cut is not the sole transcriptional regulator of Tutl since Cut is not normally detectable in class I neurons [34]. Consistent with this conclusion, we and others [28] were able to detect Tutl protein expression in *cut* mutant MARCM clones. Collectively, these results indicate that Cut is not absolutely or solely required for Tutl expression in da neurons, however this does not preclude the potential that Cut contributes to transcriptional regulation of Tutl in da neurons as our data indicate. Interestingly, ectopic expression of Cut in class I da neurons leads to a significant increase in dendritic branching complexity [34], whereas overexpression of Tutl in class I da neurons has no demonstrable effect on dendritic complexity, although our genetic interaction data reveal that Tutl is required for the observed Cut overexpression effects on class I dendritic complexity. These data suggest that Cut regulation of Tutl expression in class I neurons alone is insufficient to modulate dendritic morphology. This raises the possibility that Cut may positively regulate transcription of both Tutl and a putative partner molecule and thus the gain of function dendrite phenotype observed with ectopic Cut expression is the result of co-upregulation of both Tutl and an as yet unidentified Tutl*-*interacting protein. In support of this possibility are recent findings in which the deletion of the Tutl carboxy-terminal domain (CTD) of the longest known membrane bound Tutl isoform, RE40452, failed to produce specific loss of function defects in dendrite morphogenesis [28], whereas *tutl* mutations in general produce defects suggesting that a potential partner, possibly a co-receptor, may be required to mediate downstream signaling essential for normal class specific dendrite development. Other members of the IgSF require *cis* or *trans* interactions to perform cellular functions [40]. For example, Robo-1 interacts with DCC via conserved cytoplasmic regions to neutralize the attractive effect of Netrin-1 [41]. Furthermore, axonal repulsion following Slit binding appears to rely on interactions between Robo-1 and the intracellular phosphoprotein Enabled, which modulates cytoskeleton dynamics [42]. As Cut has been shown to modulate dendritic complexity through the actin cytoskeleton [15], it would be interesting to determine if potential cytoskeletal changes accompany *tutl* mutation. The differential changes based on neuronal class that *tutl* mutants display may manifest via different changes in the underlying dendritic cytoskeletal architecture.

### Evolutionarily conserved roles for Tutl- and Cut-related proteins in dendrite development

The *Drosophila tutl* gene shares evolutionary homology with both humans (KIAA1355) and mice (Dasm1) [31]. Our data indicate that Tutl is required for the differential regulation of class specific dendrite morphogenesis including dendritic extension and branching in class II-IV da neurons. These results are consistent with initial studies of Dasm1 which was found to be required in promoting dendrite outgrowth in cultured rat hippocampal neurons [31]. However, later studies using a *Dasm1* genetic knockout did not observe the reported morphological defects, nor any overt behavioral phenotype [33]. A possible explanation for this discrepancy is the presence of the co-expressed and closely related ortholog IgSF9b which may contribute to functional redundancy. As such, the contribution of mammalian homologs to mediating dendrite morphogenesis remains somewhat unclear. Future studies examining double knockout animals will likely be needed to fully elucidate the contribution of mammalian Tutl homologs to the regulation dendrite development. Due to its apparent lack of functional redundancy, further studies of the Tutl/Dasm1/KIAA1355 family of proteins using *Drosophila* may be a more direct route to further study this evolutionarily conserved gene family.

Similar to Tutl, evolutionarily conserved Cut-related proteins in vertebrates have likewise been implicated in regulating class-specific dendrite morphogenesis. Previous studies have demonstrated that Cut-related protein, CDP, is functionally conserved in regulating dendrite morphogenesis as expression of CDP can rescue *cut* mutant dendritic defects in *Drosophila* da neurons and ectopic expression of CDP can phenocopy dendritic defects observed with ectopic Cut expression [34]. Moreover, a recent study has implicated the Cut-related, *Cux1* and *Cux2* genes, in regulating dendritic branching, spine morphology, and synaptogenesis in upper layer neurons of the cortex [43]. Moreover, these analyses implicate Cux1 and Cux2 in regulating subclass-specific mechanisms of synapse regulation providing further evidence for the evolutionarily conserved role of Cut-related proteins in the specification of class-specific dendritic morphologies [43]. The fact that both Tutl- and Cut-related proteins display evolutionarily conserved roles in directing dendrite morphogenesis, coupled with our findings of a transcriptional regulatory interaction between Tutl and Cut, together with genetic interaction data indicating Tutl functions as a downstream of effector of Cut in da neuron dendrite morphogenesis suggests the potential that murine Cut homologs may also function in transcriptionally regulating the murine Tutl homolog, DASM1 and/or the highly orthologous IgSF9b. Ultimately, elucidating the molecular mechanisms by which Tutl and Cut mediate differential effects on dendrite development will require additional studies aimed at the identification of Tutl*-*interacting proteins which may be distinct in individual neuron subclasses and identification of other downstream effectors of Cut transcriptional regulation in da neurons.

## Materials and Methods

### *Drosophila* Strains and Culture

Fly strains used in these studies were obtained from stock centers at Bloomington (tutl^01085^, Df(2L)ed-dp, GAL4^477^,UASmCD8::GFP, FRT^40A^, Sco/CyO, DTS(1)/CyO, tubP-GAL80, Kr[If-1]/CyO; D/TM3-Ser, elavC155-GAL4,UASmCD8GFP,hsFLP; FRT^40A^,tubP-GAL80), Harvard (PBac{PB}tutl^c00018^), and other sources (tutl^ex383^ [30], tutl^23^ [28], UAS-tutl^LD28224^, UAS-tutl^GH15753^, UAS-tutl^AT02763^, UAS-tutl^HL01565^ [30], tutl-GAL4, GAL4^221^,UASmCD8::GFP, UAS-cut, GAL4^C161^,UAS-mCD8::GFP). Oregon-R was used as a wild-type strain. Drosophila stocks were raised on standard cornmeal-molasses-agar fly food medium at 25°C.

### MARCM Analyses

To conduct loss of function MARCM analyses the *tutl^c00018^* allele was recombined onto the *FRT^40A^* chromosome to generate the *FRT^40A^,tutl^c00018^* recombinant chromosome. Putative *tutl* mutant recombinant stocks were verified by genetic backcross to the parental *tutl^c00018^* allele for testing homozygous mutant lethality. Genomic DNA extracted from homozygous lethal lines were then verified for the presence of the *FRT^40A^* transgenic insertion via PCR using the following primer pair designed against the *FRT^40A^* insertion: FRT40A forward primer (5′-CACCTGCAAAAGGTCAGACA-3′) and FRT40A reverse primer (5′-CCTGACGGACCATTGATACC-3′). MARCM analyses were performed as previously described [8]. Briefly, virgin females from a stock with genotype *w,elavC155-GAL4,UASmCD8::GFP,hs-FLP; FRT40A,tub-GAL80* were crossed with males of *w; FRT^40A^,tutl^c00018^/CyO*. Embryos were collected on standard cornmeal media plates for 3 hours at 25°C, aged for 4 hours at 25°C, and heat shocked at 37°C for 1.5 hours. The plates were then stored at 25°C for 3-4 days at which point third instar larvae were screened for the presence of GFP fluorescently labeled neurons using a Leica MZ F16A stereofluorescent microscope. Third instar larvae bearing GFP labeled neurons were then subjected to live image confocal microscopy for analysis.

### Generation of anti-Tutl Antibodies

Protein sequences of all predicted *turtle* isoforms were obtained from flybase.org and analyzed by a Hopp and Woods Antigenicity Plot (JaMBW module) to predict high surface probability of putative Tutl protein antigens that lie outside of conserved domain regions of the predicted Tutl proteins (*e.g.* the immunoglobulin and fibronectin type III domains) (http://www.bioinformatics.org/JaMBW/3/1/7/) [44] and show protein sequence specificity unique to Tutl proteins and common among all predicted Tutl isoforms.. Based upon these analyses, two distinct N-terminal peptide sequences were selected for antibody production. A twenty-one amino acid peptide sequence corresponding to amino acids 73–93 (FTVKTTRRRRSRRRAEGSSIC) was used for antibody production in rabbits and an eleven amino acids peptide sequence corresponding to amino acids 22–33 (EKSKEQQQQSQ) was used for antibody production in rats. Peptides were synthesized by GenScript Corporation (Genscript, Inc. Pescataway, NJ) and coupled to KLH to improve antigenicity in peptide antibody production. Polyclonal antibodies were produced in two rabbits and two rats, respectively by Cocalico Biologicals Inc. (Reamstown, PA). IgG was isolated from the resulting rabbit and rat antisera using the NAb Protein A Plus Spin Kit (Pierce).

### Immunohistochemistry

Dissection, staining, and mounting of wandering third instar larvae was performed as previously described [8]. For immunohistochemistry (IHC), larvae were dissected to generate larval filets in cold PBS, pinned on sylgard plates and covered with a drop of 4% paraformaldehyde. Following dissection of all larvae, the plate was covered with 4% PF and incubated at RT for 20 minutes with gentle agitation. Next, larvae were washed five times in 1X PBT (PBS + 0.3% Triton X-100), the mounting pins removed, and larval cuticles transferred to 6×50 mm borosilicate glass tubes (Kimble Glass, Inc.) pre-coated with 1X PBT to reduce surface adhesion of the larval cuticles to the walls of the glass tubes. Larvae were blocked in 5% normal donkey serum (Jackson Laboratories, West Grove, PA) for at least 30 minutes at RT or overnight at 4°C. Primary antibodies used in these studies include: rabbit anti-Tutl (1∶1000), rat anti-Tutl (1∶100), mouse anti-CD8 (1∶100) (Invitrogen), rabbit anti-EGFP (1∶2000) (Clontech, Inc.), and mouse anti-Cut (2B10; 1∶50) (Developmental Studies Hybridoma Bank). Donkey anti-rat, anti-rabbit, and anti-mouse secondary antibodies were used at 1∶200 (Jackson Immunoresearch). Filets were either mounted on poly-L lysine-coated coverslips followed by ethanol dehydration, xylene clearing, and DPX mounting or alternatively were mounted directly onto coverslips under a drop of glycerol. IHC slides were then imaged on a Nikon C1 Plus confocal microscope.

### Confocal Microscopy and Live Imaging

For live image analyses, third instar larvae were placed on a clean glass microscope slide, immersed in a few drops of 1∶5 (v/v) diethyl ether to halocarbon oil and covered with a 22×50 cm glass coverslip. Neurons expressing GFP were visualized using a Nikon C1 plus confocal microscope using the Nikon EZ-C1 software. Confocal images were collected as a series of z stacks with a depth of 1.5 µm and 1024×1024 pixel resolution. Z-stacks were then rendered into a maximum projection and resultant images were processed for quantitative neuronal reconstruction analyses.

### Neuronal Reconstruction, Morphometric Data Analyses and Statistics

Representative neurons from loss of function and gain of function analyses were selected for quantitation based on the quality of the 2D confocal projection images and the absence of disrupted dendritic branches. Quantification of dendritic arbor complexity from representative neurons was performed by collecting z-series images acquired on a Nikon C1 Plus confocal microscope using a 20X (0.75 N.A.) objective, projected into a 2D image and imported into the Neuromantic software package for generation of neuronal reconstructions (.swc files) (http://www.reading.ac.uk/neuromantic/). Reconstruction files (.swc) were then input into the L-Measure software package [45] (http://cng.gmu.edu:8080/Lm/) and assigned parameters including total dendritic length, number of terminals and dendritic branch order. Statistical analyses, including means ± SD, were performed on both individual neurons and classes of da neurons where both control and mutant have an *n* value equal to 5 or more. Statistical calculations were performed by importing data generated in L-measure into SigmaPlot (Systat Software) in which Student's *t*-test, Wilcoxon/Mann-Whitney rank sum *t*-tests or two-way ANOVA analyses were conducted depending upon sample size and complexity. Both wild-type and *tutl* mutant neuronal reconstruction files were submitted to NeuroMorpho.org (http://neuromorpho.org) for digital curation.

To assess relative expression levels of the *tutl-GAL4,UASmCD8::GFP* reporter strain and Tutl immunoreactivity in da neuron subclasses, third instar larvae expressing the *GAL4* reporter or labeled with anti-Tutl antibodies were imaged by collecting z-stack confocal micrographs. Identical laser power and gain settings were used to assess wild-type control and experimental samples. Individual 1 µm z-sections were projected into 2D images which were then imported into Photoshop (Adobe) for pixel density measurements. To quantify pixel intensity, polygons were drawn around each cell body and the histogram function was used to determine the average intensities and the total pixels. These values were used to calculate the average intensity per area by dividing the average intensity values by the total pixels for each cell body.

### Chromatin Immunoprecipitation (ChIP)

ChIP was performed using the EZ Chip^TM^ Kit (Millipore, Inc. Temecula, CA). Briefly, S2 cells were grown in a 75 cm^2^ T-flasks to a density of 5×10^6^ in 25 ml of Schneider's *Drosophila* medium supplemented with 10% fetal bovine serum. Chromatin was crosslinked by the addition of 675 ul of 37% formaldehyde (1% final concentration). Following incubation at room temperature for 10 minutes, cells were dislodged, transferred to centrifuge tubes, and resuspended in lysis buffer. Cell lysates were then sonicated with a Misonix-4000 (Qsonica, LLC. Newton, CT) on ice at 15% power for 4 pulses of 10 seconds on and 30 seconds off. This sonication protocol produced DNA fragments between 200–800 base pairs.

The lysates were then precleared with protein G sepharose beads. Following this step, 10 µl was removed from each treatment and used for input control. Lysates were then incubated overnight with either 10 µg of anti-Cut antibody (2B10) or 5 µg of normal mouse IgG antibody as a negative control from the kit. Crosslinks were then reversed and DNA was isolated for analysis. Immunoprecipitated DNA from both experimental and control samples was analyzed with real-time PCR using a Bio-Rad iCycler IQ and iQ Sybr Green Supermix (Bio-Rad Laboratories, Inc. Hercules, CA). The following primer pair flanking the genomic target sequence of interest was used for detection of the *tutl* promoter region containing the Cut URE binding site: Tutl-URE forward primer (5′-ATTTCAATCGCAGCGTTCAGTGGC-3′) and Tutl-URE reverse primer (5′-TAGATTTGGATCTGGGCACGT-3′). The following primer pair specific to the GAPDH2 gene was used as a negative control for the Cut ChIP experiment: GAPDH2-F (5′GCTCATATATGTGTATTCGTGTGCC-3′) and GAPDH2-R (5′-ACTTCTGATCTTAGACAGTTAAAGTGACC-3′). CT values were computed by MyiQ®software (Bio-Rad Laboratories, Inc. Hercules, CA).

### Cell Isolation and qPCR

For analyses of Cut transcriptional regulation of *tutl*, RNA was purified from class I da neurons of the following genotypes: (1) *w; UAS-cut; GAL4^221^,UASmCD8::GFP* (experimental; ectopic Cut expression in class I da neurons) and (2) +; +; *GAL4^221^,UASmCD8::GFP* (control; wild-type class I da neurons). Class I da neuron RNA from these two genotypes was isolated via magnetic bead based cell sorting essentially as described in Iyer *et al.* (2009). Following RNA purification from isolated class I da neurons, qPCR was performed using QuantiTect Primer Assays with real-time primers optimized for *tutl* (Qiagen, Cat. No. QT00502537) and *cut* (Qiagen, Cat. No. QT00501389). These optimized and pre-validated primers allow for qPCR against all *tutl* isoforms and the *cut* homeodomain transcription factor.

## Supporting Information

Figure S1 ***tutl*** **mutation results in a proximal shift of branch order in class IV da neurons.** Graph representing the percentage of branches from each class IV ddaC neuron with a given branch order. Trend lines represent the moving average. Vertical lines indicate highest frequency of branches. Note that *tutl* mutants display a proximal shift toward lower order branches as compared to wild-type controls.
(TIF)

Figure S2 **Dendritic self-avoidance is unaffected in** ***tutl*** **mutant class IV da neurons.** (**A**) Quantification of the total number of dendritic crossovers in class IV da neuron MARCM clones from wild-type (WT; n = 10) and *tutl^c00018^* (n = 11). Relative to wild-type, the number of dendritic crossovers in *tutl* mutant clones was not significant (n.s.) (*t-*test, *p* = 0.541). (**B**) Quantification of the total number of dendritic crossing points normalized to total dendritic length in class IV da neuron MARCM clones from wild-type (WT; n = 10) and *tutl^c00018^* (n = 11). Relative to wild-type, the normalized number of dendritic crossovers in *tutl* mutant clones was not significant (n.s.) (*t*-test, *p* = 0.289). (**C**) Representative image of wild-type class IV ddaC MARCM clone where dendritic crossovers are indicated by numbers (red). (**D**) Representative image of *tutl^c00018^* mutant class IV ddaC MARCM clone where dendritic crossovers are indicated by numbers (red).
(TIF)

Figure S3 **Isoform-specific Tutl overexpression has no effect on class I da neuron dendrite development.** Overexpression of four distinct Tutl isoforms (*AT02763, GH15753, HL01565,* and *LD28224*) in class I da neurons has no significant (n.s.) effect on either overall dendritic length (**A**) or dendritic branching complexity measured by the number of dendritic terminals (**B**) as compared to wild-type controls (Student's *t*-test, *p*>0.05). The total *n* value for each neuron subtype is indicated on the bar graph. Statistical analyses were performed pair-wise between wild-type controls and each of the Tutl isoforms. Genotypes: **WT**: *GAL4^221^,UASmCD8::GFP/+*; **AT02763**: *UAS-tutl^AT02763^/+; GAL4^221^,UASmCD8::GFP/+*; **GH15753**: *UAS-tutl^GH15753^/+; GAL4^221^,UASmCD8::GFP/+*; **HL01565**: *GAL4^221^,UASmCD8::GFP/UAS-tutl^HL01565^*; **LD28224**: *GAL4^221^,UASmCD8::GFP/UAS-tutl^LD28224^*.
(TIF)

Table S1 ***tutl^c00018^*** **complementation analyses.** Trans-heterozygotes *tutl^c00018^* allele with the previously characterized *tutl^01085^* allele [27] and *tutl* deficiency stock (*Df(2L)ed-dp*) reveal a complete failure to complement as indicated by the 100% trans-heterozygous lethality. *n* represents the total number of progeny examined from each complementation cross.
(DOC)

Table S2 ***tutl^c00018^*** **rescue analyses.** Pan-neuronal expression of the *UAS-tutl^AT02763^* transgene via *elavGAL4* completely rescues adult viability of *tutl^c00018^* homozygous mutant females. As the *UAS-tutl^AT02763^* and *elavGAL4,UASmCD8::GFP* transgenes both map to the X chromosome, only females in this rescue experiment will inherit one copy of each transgene. *n^obs^* represents the number of adults observed whereas *n^exp^* represent the number of adults expected for rescue. Rescue is reported as N.A. (not applicable) for *tutl^c00018^/CyO* heterozygous females which are viable in the presence or absence of neuronal expression of the *UAS-tutl^AT02763^* transgene.
(DOC)
