# Supplementary figures and images for: Turtle Functions Downstream of Cut in Differentially Regulating Class Specific Dendrite Morphogenesis in Drosophila
Source: PLoS One. 2011 Jul 21;6(7):e22611. doi: 10.1371/journal.pone.0022611 (PMC3141077; doi:10.1371/journal.pone.0022611)

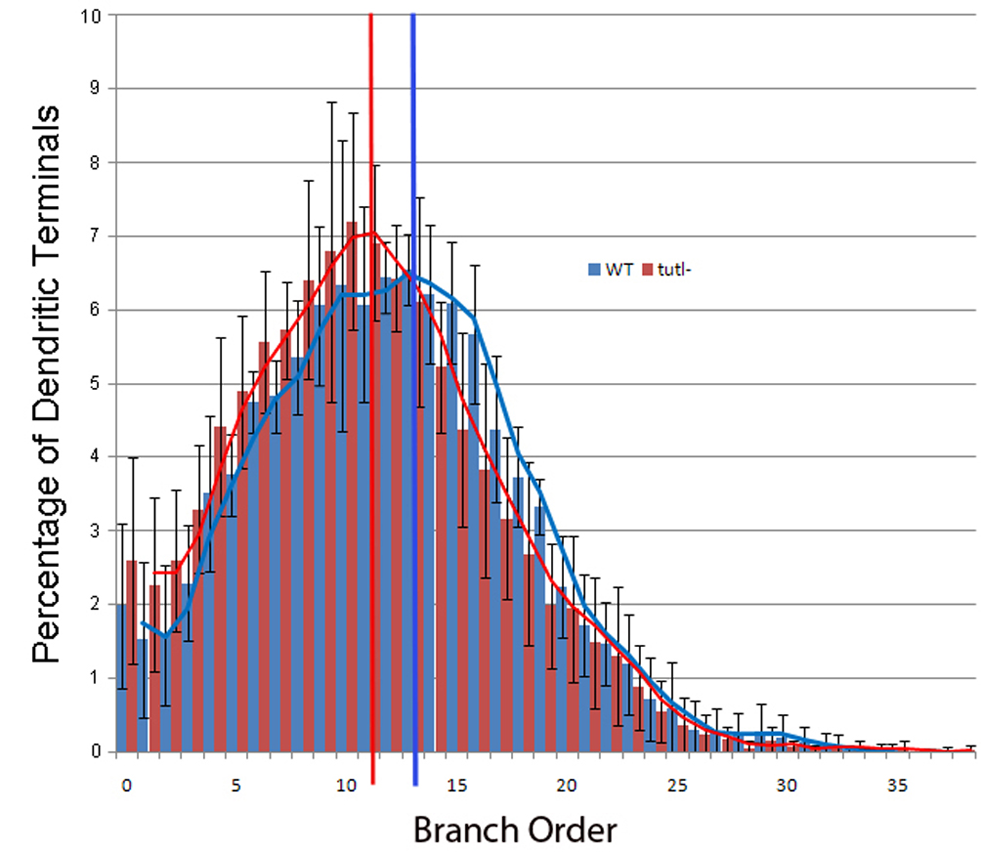

Supplement: Figure S1 — tutl mutation results in a proximal shift of branch order in class IV da neurons. Graph representing the percentage of branches from each class IV ddaC neuron with a given branch order. Trend lines represent the moving average. Vertical lines indicate highest frequency of branches. Note that tutl mutants display a proximal shift toward lower order branches as compared to wild-type controls. (TIF) [file pone.0022611.s001.tif]

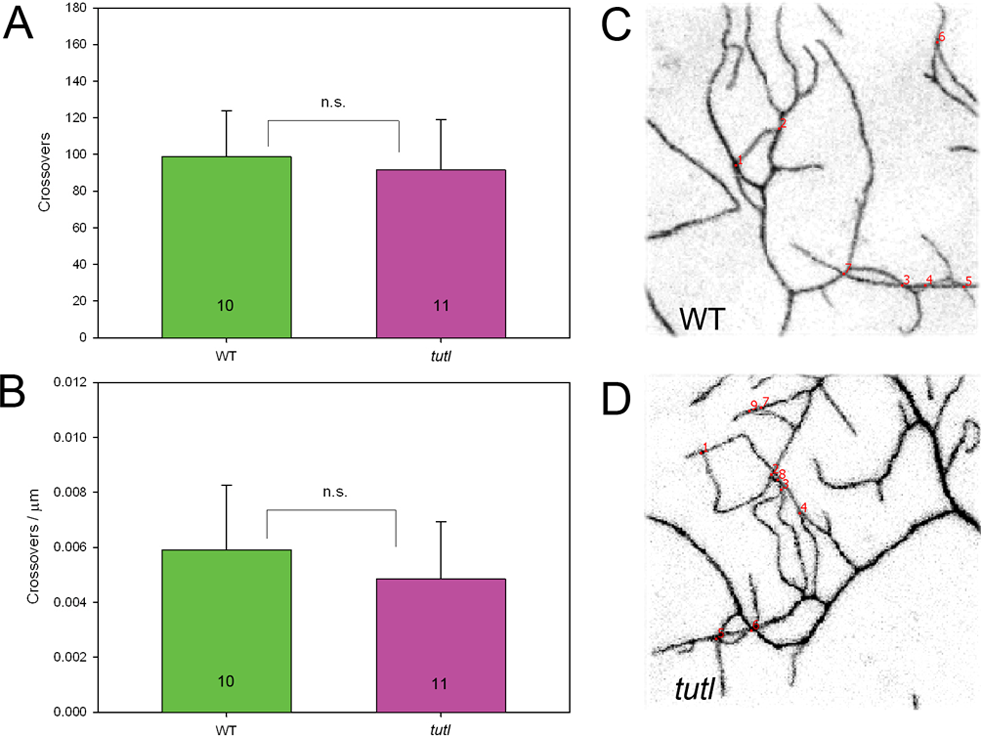

Supplement: Figure S2 — Dendritic self-avoidance is unaffected in tutl mutant class IV da neurons. (A) Quantification of the total number of dendritic crossovers in class IV da neuron MARCM clones from wild-type (WT; n = 10) and tutlc00018 (n = 11). Relative to wild-type, the number of dendritic crossovers in tutl mutant clones was not significant (n.s.) (t-test, p = 0.541). (B) Quantification of the total number of dendritic crossing points normalized to total dendritic length in class IV da neuron MARCM clones from wild-type (WT; n = 10) and tutlc00018 (n = 11). Relative to wild-type, the normalized number of dendritic crossovers in tutl mutant clones was not significant (n.s.) (t-test, p = 0.289). (C) Representative image of wild-type class IV ddaC MARCM clone where dendritic crossovers are indicated by numbers (red). (D) Representative image of tutlc00018 mutant class IV ddaC MARCM clone where dendritic crossovers are indicated by numbers (red). (TIF) [file pone.0022611.s002.tif]

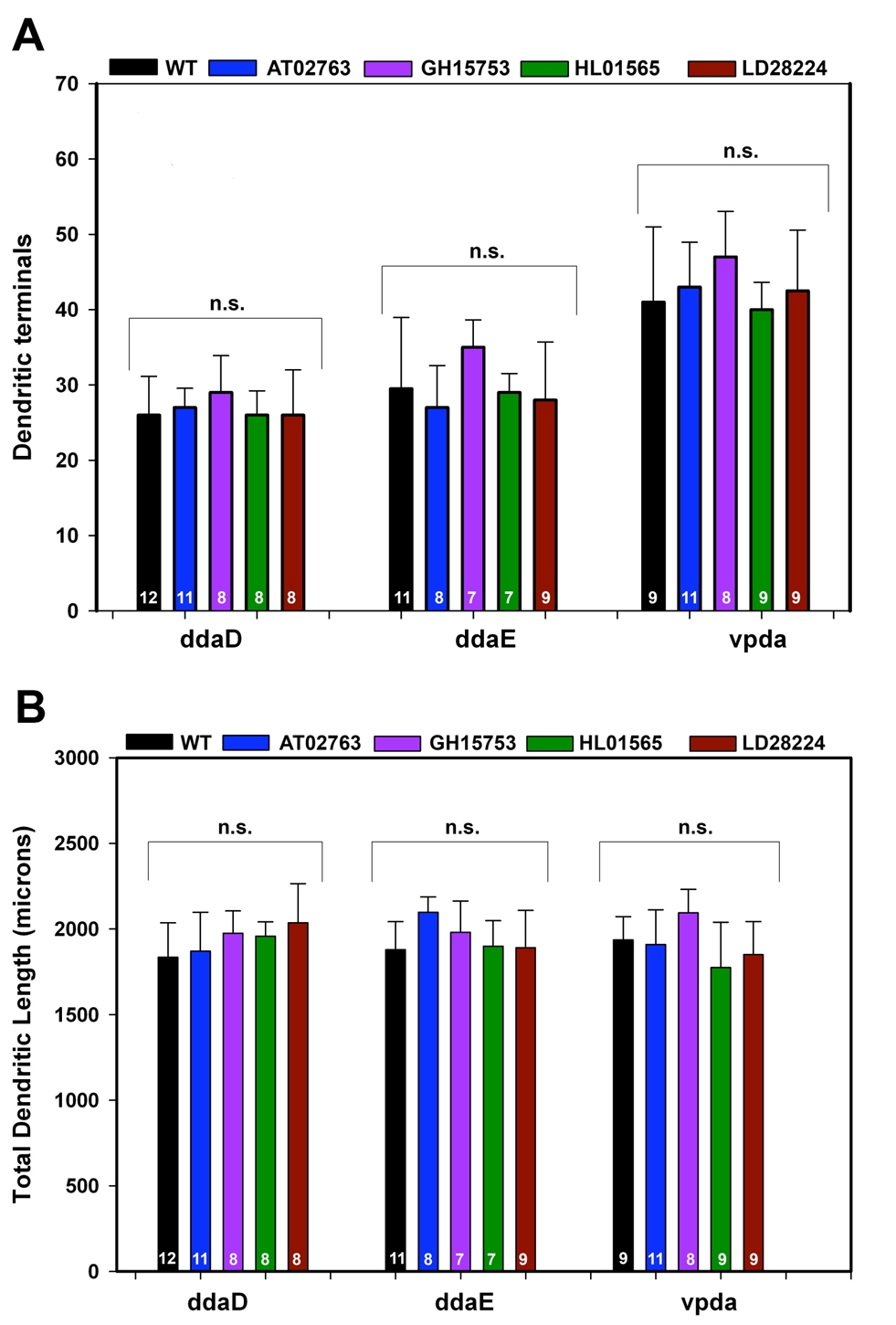

Supplement: Figure S3 — Isoform-specific Tutl overexpression has no effect on class I da neuron dendrite development. Overexpression of four distinct Tutl isoforms (AT02763, GH15753, HL01565, and LD28224) in class I da neurons has no significant (n.s.) effect on either overall dendritic length (A) or dendritic branching complexity measured by the number of dendritic terminals (B) as compared to wild-type controls (Student's t-test, p>0.05). The total n value for each neuron subtype is indicated on the bar graph. Statistical analyses were performed pair-wise between wild-type controls and each of the Tutl isoforms. Genotypes: WT: GAL4221,UASmCD8::GFP/+; AT02763: UAS-tutlAT02763/+; GAL4221,UASmCD8::GFP/+; GH15753: UAS-tutlGH15753/+; GAL4221,UASmCD8::GFP/+; HL01565: GAL4221,UASmCD8::GFP/UAS-tutlHL01565; LD28224: GAL4221,UASmCD8::GFP/UAS-tutlLD28224. (TIF) [file pone.0022611.s003.tif]
