# Supplementary material for: Turtle Functions Downstream of Cut in Differentially Regulating Class Specific Dendrite Morphogenesis in Drosophila
Source: PLoS One. 2011 Jul 21;6(7):e22611. doi: 10.1371/journal.pone.0022611 (PMC3141077; doi:10.1371/journal.pone.0022611)
Supplement: Table S1 — tutlc00018 complementation analyses. Trans-heterozygotes tutlc00018 allele with the previously characterized tutl01085 allele [27] and tutl deficiency stock (Df(2L)ed-dp) reveal a complete failure to complement as indicated by the 100% trans-heterozygous lethality. n represents the total number of progeny examined from each complementation cross. (DOC) [file pone.0022611.s004.doc]

| **Genotype** | ***n*** | **% Lethality** |
| --- | --- | --- |
| *tutl^c00018^/tutl^c00018^* | 53 | 100% |
| *tutl^c00018^/tutl^01085^* | 41 | 100% |
| *tutl^c00018^/Df(2L)ed-dp* | 58 | 100% |
