# Supplementary material for: Turtle Functions Downstream of Cut in Differentially Regulating Class Specific Dendrite Morphogenesis in Drosophila
Source: PLoS One. 2011 Jul 21;6(7):e22611. doi: 10.1371/journal.pone.0022611 (PMC3141077; doi:10.1371/journal.pone.0022611)
Supplement: Table S2 — tutlc00018 rescue analyses. Pan-neuronal expression of the UAS-tutlAT02763 transgene via elavGAL4 completely rescues adult viability of tutlc00018 homozygous mutant females. As the UAS-tutlAT02763 and elavGAL4,UASmCD8::GFP transgenes both map to the X chromosome, only females in this rescue experiment will inherit one copy of each transgene. nobs represents the number of adults observed whereas nexp represent the number of adults expected for rescue. Rescue is reported as N.A. (not applicable) for tutlc00018/CyO heterozygous females which are viable in the presence or absence of neuronal expression of the UAS-tutlAT02763 transgene. (DOC) [file pone.0022611.s005.doc]

| **Genotype** | ***n^obs^*** | ***n^exp^*** | **% Rescue** |
| --- | --- | --- | --- |
| *elavGAL4,UASmCD8::GFP/UAS-tutl^AT02763^; tutl^c00018^/tutl^c00018^* | 19 | 14 | 100% |
| *elavGAL4,UASmCD8::GFP/UAS-tutl^AT02763^; tutl^c00018^/CyO* | 24 | 29 | N.A. |
